# An In-Depth Prospective Comprehensive View on Myocardial Infarction (MI) in Young Adults

**DOI:** 10.7759/cureus.40630

**Published:** 2023-06-19

**Authors:** Usha Topalkatti, Madhusudhan Chennamalla, Ramjoshna N, Paramesh B, Rajarahulnaik Banothu

**Affiliations:** 1 Internal Medicine, Spartan Health Sciences University School of Medicine, Vieux Fort, LCA; 2 Radiology, Mediciti Institute of Medical Sciences, Hyderabad, IND; 3 Pulmonary Medicine, Mediciti Institute of Medical Sciences, Hyderabad, IND; 4 Internal Medicine, Mediciti Institute of Medical Sciences, Hyderabad, IND

**Keywords:** coronary heart disease, young people, women, modifiable risk factors, myocardial infarction

## Abstract

Due to major advancements in myocardial infarction (MI) prevention and effective medical treatment, the death rate and incidence of MI have dropped considerably. We know that their risk factors and prognosis may differ; therefore, increasing primary and secondary prevention activities among young people is crucial. Multiple studies have found that MI is the deadliest form of coronary heart disease (CHD). As a result, we made an effort to illuminate MI in young people in our review of the literature. We found that young people, particularly women, are developing MI. Smoking is a key risk factor that should be targeted in an effort to minimize youth MI rates. It is thus important to create superior methods for measuring risk in young people, which may combine both standard and nonconventional risk factors, such as genetic rate scores and coronary artery calcium testing. Henceforth, addressing modifiable risk factors at a younger age has the greatest impact.

## Introduction and background

The mortality rate and incidence of myocardial infarction (MI) have decreased due to significant advancements in MI prevention and the utilization of efficacious medical therapy. However, the rates of MI in younger people, especially among women, have not decreased in a similar way as that for the older ones [1-3]. Therefore, this makes it crucial for increasing primary and secondary prevention efforts among young individuals because we know that their risk factors and prognoses may differ from each other [4-7]. Furthermore, research has shown that MI in younger people might have more severe outcomes since they have a higher economic and societal impact [8]. When it comes to the term 'young', numerous studies indicate that individuals between ages 30 and 55 are considered to be in the 'young' age bracket [8]. Furthermore, many studies have concluded that MI is the lethal manifestation of coronary heart disease (CHD). Hence, in this review paper, we have attempted to shed some light on MI in young adults.

## Review

Risk factors

Many past studies suggest that there are multifactorial trends present regarding the higher proportion of MI in younger adults [9]. Insufficient data exist regarding cardiovascular (CV) disease in young individuals, leading to a lack of recognition in this population [8]. The occurrence of multiple crucial controllable factors for risk is increasing [8]. For several decades, numerous studies have identified the use of tobacco as a prominent and widespread risk factor among young patients who present with MI [7,9]. In this respect, one study found that patients in the age group between 18 and 49 years for both men and women randomly smoked and concluded that they were at risk of developing MI by approximately ninefold and 13-fold, respectively, compared to those who are non-smoking [10]. The utilization of cocaine and marijuana has been observed to be notably widespread among young patients who encounter MI, as per various previous studies [5,11]. A comparable investigation was conducted to substantiate the aforementioned assertion, in which the author endeavoured to assess the prevalence of cocaine or marijuana consumption among more than 10% of young adults (under 50 years of age) who presented themselves as MI patients at their medical facility during the study duration spanning from 2000 to 2016. The researchers arrived at the conclusion that all of the factors under consideration were linked to a rise in both overall mortality (HR 1.99, p=0.001) and CV mortality (HR 2.22, p=0.005) during a median follow-up duration of 11.2 years [5].

Studies have proved that the lifestyle that we have today enables an increase in the risk of obesity and diabetes even in young adults [7]. The study by Yandrapalli et al. found that more people are at risk due to obesity up to 98% [7]. Similarly, some studies also found that the prevalence of metabolic syndrome in young individuals had increased nowadays, as nearly half of young MI patients had metabolic syndrome [12,13]. Furthermore, diabetes is another risk factor that is nowadays highly prevalent in young adults. Studies conducted in the past have found that one in five young MI patients showed the symptoms of and had diabetes mellitus at the time of their reporting and admission to the hospital for their treatment. Due to this, patients have also a higher long-term risk (HR 1.65, p=0.008) and CV mortality (HR 2.10, p=0.004) [6]. Several studies have indicated that a variety of unmodifiable risk factors significantly influence the development of MI in young individuals. The aforementioned factors encompass genetic predispositions, such as heightened lipoprotein levels, familial hypercholesterolemia (FH), and polygenic risk. These may be prevalent among younger patients who experience their initial MI at an early age [14]. Due to the relatively lower prevalence of conventional CV risk factors among younger patients compared to older individuals, the role of non-traditional risk factors in forecasting a future MI in the younger population may be of particular significance [15-17].

According to previous research, elevated Lp(a) levels are more common in young people who have had MI [14]. A recent study demonstrated significant risk ratios of 1.16 and 1.18, correspondingly for coronary artery disease (CAD) and MI, with an increase of 50 nM in Lp(a) levels (p<0.0001). Elevated levels of Lp(a) not only serve as a risk factor for MI but also possess a robust predictive capacity for anticipating unfavourable outcomes in individuals with a prior history of MI [8]. Lp(a) levels of more than 50 mg/dL were shown to be substantially linked with an elevated risk of future CV events in MI patients with pre-diabetes or diabetes (HR 2.7, p=0.05 for pre-diabetes and HR 3.5, p=0.05 for diabetes) [18]. FH has been recognized as a significant genetic predisposition for the occurrence of MI in young adults since ancient times. Additionally, a YOUNG-MI Registry study demonstrated that approximately 10% of individuals under the age of 50 who experienced MI had a clinically established association with FH. Studies have also shown that patients with FH had a future risk of developing CV [17]. Findings have also concluded that there is an important link between CV and FH patients [19]. Consequently, all the previous trials underscored the necessity of implementing more assertive cholesterol-lowering therapy for individuals with FH to prevent primary MI.

'Polygenic risk' has recently gained popularity and interest, especially for young adult patients with genetically developing CAD [20,21]. Previous studies have indicated that the evaluation of an individual's genetic code, particularly CAD-risk-associated single nucleotide polymorphisms, which remain constant from birth, can directly determine this type of risk. This finding has been documented in various research studies [22]. In addition, it has been observed that conventional risk calculators may not provide a precise estimation of CV risk in the younger population [17]. However, the utilization of polygenic risk scores has the potential to enhance the categorization of risk in this cohort [8].

Classification

Yandrapalli et al. found that, among young patients (aged 18-44) with MI, the prevalence rates were as follows: smoking (56.8%), dyslipidemia (51.7%), and hypertension (49.8%) [8].

Pathogenesis

The pathogenesis of MI involves various causes that have been classified into four categories. The first category is atheromatous CHD. The second category is non-atheromatous coronary artery abnormalities. The third category is hypercoagulable states that involve conditions characterized by increased blood clotting tendencies. The fourth category is MI related to substance misuse, which encompasses cases where substance abuse, such as cocaine or amphetamines, contributes to the development of MI [21].

Atheromatous CAD

As per scholarly research, the atheromatous process initiates at an early stage of life. A study conducted on 760 young adult patients who passed away due to various causes revealed that 20% of males and 8% of females aged between 30 and 34 years had advanced CHD, according to a necropsy examination [22]. The Bogalusa Heart Study and Pathological Determinants of Atherosclerosis in Youth investigation may exhibit analogous trends [23,24]. Furthermore, typical risk factors for this type, such as cigarette smoking, were shown to be present in up to 92% of young patients in numerous investigations for atheromatous processes [25]. The study findings indicate that individuals below the age of 40 years exhibited a higher prevalence of smoking compared to those aged 60 years and above, among patients who had undergone percutaneous coronary intervention (PCI). The statistical analysis revealed a significant difference between the two groups with a p-value of 0.01 [26]. According to a study conducted in London, it was found that 39% of young patients with MI had a positive family history of early CHD [27]. Offspring of parents with early onset of CHD exhibit a greater incidence of lipid abnormalities, insulin resistance, and obesity, indicating a plausible genetic correlation. These folks had greater vascular anomalies than patients who had MI before the age of 45 [22].

Non-atheromatous Coronary Artery Abnormalities

Acute MI (AMI) may be the first symptom of congenital coronary artery abnormalities in young individuals. They are so uncommon that the cardiologist performing the catheterization may be surprised. In certain cases of myocardial bridging, which has been observed in young patients with MI, the coronary arteries may be discovered in a tunnel in the heart beneath a layer of muscles. An MI may occur as a result of myocardial bridging because of severe ischaemia caused by the bridging during systolic contraction. PCI or surgical splitting was demonstrated to be more beneficial than pharmacological therapy for these patients. MI in younger people has been reported as a consequence of septic vegetation from an infected aortic valve. Users who inject drugs are at greater risk [28]. It has been reported that, due to a lack of vegetation, young people may obtain MI as a result of bacteremia [21]. The aetiology of sepsis is often addressed as an integral component of the therapeutic regimen. MI in young individuals is rarely caused by coronary artery aneurysms. The prevailing theory is that MI occurs due to either embolization from the aneurysmal sac or extraluminal compression [29]. The occurrence of MI has been noted in cases where there has been right-to-left embolization through a patent foramen ovale [30,31].

Hyper-Coagulable States

Arterial and venous thrombosis occurs repeatedly, which is associated with antiphospholipid syndrome. Most people in their 30s who get the disease are young. Other autoimmune disorders, such as systemic lupus erythematosus, might be primary or secondary associated with it. AMI may occur when a coronary artery is blocked by a clot [32]. Premature atherosclerosis and elevated platelet adhesion are common in these individuals [21]. Antiphospholipid antibody titres are not always associated with disease activity, and a thorough assessment is advised for those who are doubtful [21]. The hypercoagulability associated with nephrotic syndrome is caused by a combination of variables, such as fibrinolytic system abnormalities, dyslipidemia, and a decrease in anticoagulant factors. The majority of individuals' thrombophilia might be attributed to a deficiency of antithrombin III, a coagulation inhibitor [33].

Clinically MI Patients

It is recommended that a thorough assessment of recent recreational drug usage be conducted for all young patients who present with MI. The presence of a family history of early onset of CHD and a risk factor profile that encompasses smoking, obesity, diabetes, and dyslipidemia are considered to be more reliable indicators for predicting the likelihood of atheromatous CAD. It is recommended to disclose any prior occurrences of venous or arterial thrombosis. The foremost clinical consideration is to ascertain hemodynamic stability, indications of sympathetic hyperactivity (e.g. tachycardia and sweating), and prior administration of injectable medication. It is recommended that individuals undergo screening for dyslipidemia stigmata, including xanthelasma, arcus senilis, and tendon xanthomata. Prompt presentation to the emergency department, subsequent to the commencement of thoracic discomfort, is often correlated with ST-segment elevation on the electrocardiogram (ECG). Serial ECGs are highly informative in relation to cocaine use due to their association with dynamic ST-segment elevation. Upon the application of vasodilators to individuals experiencing coronary artery spasms caused by cocaine consumption, both thoracic discomfort and electrocardiogram irregularities reverted to a standard state [34]. Patients presenting with chest pain beyond 12 hours from the onset are more likely to manifest abnormal Q waves. Individuals with partially occluded coronary arteries are also found to exhibit non-specific T-wave abnormalities, ST depression, and T-wave inversion. The presence of pleuritic chest pain accompanied by concave upward ST-segment elevation in the lateral leads may indicate the occurrence of myopericarditis in the patient [21].

Management 

The initial management of MI in younger patients exhibits minimal deviation from the standard management approach employed in adult patients. The first-line treatments for patients are suggested to include oxygen, diamorphine, nitrates, and aspirin. The occurrence of sudden coronary spasms and the paradoxical worsening of chest pain can be attributed to a stimulus [21]. Therefore, it is recommended that individuals with a history of cocaine use refrain from using B-blockers for a period of 48 hours. Benzodiazepines are recommended as the initial treatment for MI in individuals who use cocaine. To prevent additional coronary spasms, it is recommended to sustain nitrates in these patients [32]. It is also recommended that individuals experiencing hemodynamic instability receive early coronary angiography and intervention, and it is advisable to seek professional guidance promptly.

Patients should be given thrombolytic treatment if nitrates do not reduce cocaine-induced persistent ST elevation. When ECGs indicate good ST-segment resolution, younger people tend to tolerate thrombolytic medicines better. Risk stratification in patients with non-ST segment elevation MI should be conducted subsequent to the initial medication administration, taking into account persistent or dynamic ECG abnormalities, a more significant degree of cardiac enzyme elevation, and the presence of supplementary risk factors such as diabetes mellitus. The need for prompt coronary angiography and intervention in individuals at high risk should be assessed by referring them to specialists. Because many younger patients have completely healthy coronary arteries, not all are offered coronary angiography. Exercise stress testing may be effective for risk classification in those who have already had a MI. The significant majority of young patients who completed stage three of the Bruce regimen (nine minutes or longer) had normal coronary arteries. In all patients, echocardiography should be used to assess the left ventricular function [21].

Prevention

Elevated blood pressure and cholesterol levels among young individuals may warrant early intervention and management. Nevertheless, it is imperative to introduce lifestyle modifications for individuals across the board. Examples of healthy lifestyle choices include refraining from tobacco use, engaging in consistent physical activity, achieving and maintaining a healthy body weight, and consuming a diet rich in whole, plant-based food while limiting the intake of processed food and added sugars. A diet high in plant-based food may help those at risk of diabetes and those who are overweight in maintaining healthy blood sugar levels. Helping people stop using drugs and alcohol should be taken more seriously [35]. It is noteworthy to acknowledge the constraints of the pooled cohort equation calculators. However, it is imperative to emphasise that, irrespective of the 10-year atherosclerotic CV disease score, five lifestyle modifications must be incorporated [21].

The therapeutic benefits of statins go beyond reducing cholesterol levels and are thus recommended for all MI patients. In patients with atheromatous CHD, statins are thought to stabilise plaques, improving their prognosis and reducing recurrent occurrences. Additional medications such as niacin and omega-3 fatty acids should be investigated in rare cases such as hypertriglyceridemia and low HDL values [36,37].

Weinberger et al. conducted a study to assess and delineate the clinical characteristics and progression of patients who were admitted to a hospital for acute MI within the past 15 years and were aged 30 years or younger [38]. The researchers arrived at the conclusion that smoking has emerged as the primary risk factor for coronary disease, whereas the incidence of other conventional risk factors is relatively low. The preceding acute MI was not significantly influenced by either physical exertion or angina pectoris. Elevated levels of aggregated platelets in the circulation may have a potential pathogenic impact on patients with MI, particularly in the age group below 30 years. Similarly, Walkotte et al. conducted a study to analyse the angiographic features, coronary risk factors, and future outlook of young male and female individuals who had previously experienced MI compared to their older counterparts [39]. The researchers arrived at the conclusion that the prognosis of young patients suffering from MI is comparatively favourable compared to that of older patients.

Osula et al. carried out a case report positing that the occurrence of acute MI in a young adult can plausibly be attributed to arterial thrombosis [34]. This thrombosis is likely a result of a hypercoagulable state that arises from the nephrotic syndrome. The authors arrived at the conclusion that meticulous modification of risk factors and appropriate treatment of the root cause may lead to a decrease in the frequency of recurrent cardiac events. Bajaj et al. also conducted a study with the aim of presenting a comprehensive analysis of the similarities and differences observed in younger and older patients who were diagnosed with AMI [40]. The ultimate objective of this study was to utilise the findings as a tool to facilitate primary and secondary prevention measures in the future. The researchers arrived at the conclusion that there are significant differences in the risk factor profile and angiographic involvement among high-risk younger adults. This underscores the importance of implementing a proactive strategy aimed at preventing premature CV disease through primary and secondary prevention measures. Further, Incalcaterra et al. conducted a study to assess the risk profile and determinants of outcomes among these patients, which enables the implementation of preventive interventions [41]. The researchers arrived at the conclusion that MI in young adults exhibits distinct characteristics, not only in terms of risk factors but also in angiographic manifestation and prognosis [41].

Bhardwaj et al. conducted a study to assess the risk factors and angiographic characteristics of young individuals who have experienced AMI [42]. The findings indicate that AMI exhibits a higher incidence rate among the male population in their youth, with ST elevation MI being the most commonly observed manifestation. The predominant form of MI is the anterior wall MI, wherein the left anterior descending artery (LAD) is involved in approximately 66% of cases. The primary determinants of risk comprise smoking, hypertension, low levels of HDL, and elevated triglyceride concentrations. Zeitouni et al. undertook research to assess how guideline revisions influenced the identification of young people with early MI for preventive treatment [43]. Gao et al. also undertook research to examine and compare the characteristics of male adults who first had AMI and to investigate the relationship between age and clinical outcomes [44]. They concluded that males under the age of 50 who had their first AMI had a decreased death risk. They also carried a greater load of modifiable conventional risk factors. As a result, all young AMI patients should have their modifiable lifestyles managed.

Lu et al. conducted research to assess and compare effective prevention, which requires awareness of the risk variables linked with the risk of AMI in young women vs. men [45]. They found that there were statistically significant variations in risk factor profiles and risk factor relationships based on gender and AMI subtype. Sawano et al. conducted research on adults aged 18-55 years to investigate and establish sex differences in the causes and timing of one-year outcomes following AMI. They concluded that young women with AMI have worse outcomes than men in the year following their discharge [46]. As a result, coronary-related hospitalisations were the most prevalent, whereas noncardiac hospitalisations had the greatest sex discrepancy.

## Conclusions

MI is becoming more common among young adults. Smoking is a major risk factor and should be the focus of any programme aimed at lowering the rate of MI among the ‘young.’ These individuals frequently lack warning signs of increasing chest discomfort. In actuality, young individuals, particularly young women, contribute to an increasing proportion of MI hospitalisations. This demonstrates the importance of tailoring existing preventative approaches to target risk factors linked to CV disease in young individuals. Furthermore, new techniques for risk assessment in young individuals are necessary, which will most likely include both classic and novel risk indicators, such as genetic risk scores and, in certain situations, coronary artery calcium testing. Treatment of avoidable risk factors, in particular, should begin at a younger age.
